# Metabolic Pathway Profiling in Intracellular and Extracellular Environments of *Streptococcus thermophilus* During pH-Controlled Batch Fermentations

**DOI:** 10.3389/fmicb.2019.03144

**Published:** 2020-01-21

**Authors:** Yali Qiao, Gefei Liu, Xuepeng Lv, Xuejing Fan, Yanjiao Zhang, Li Meng, Mingzhi Ai, Zhen Feng

**Affiliations:** Key Laboratory of Dairy Science, Ministry of Education, College of Food Science, Northeast Agricultural University, Harbin, China

**Keywords:** *Streptococcus thermophilus*, metabolic pathway, intracellular metabolite, extracellular metabolite, pH-controlled batch fermentations

## Abstract

Elucidating the metabolite profiles during the growth of *Streptococcus thermophilus* is beneficial for understanding its growth characteristics. The changes in the intracellular and extracellular concentrations of carbohydrates, nucleotides, amino sugars, nucleoside sugars, fatty acids, and amino acids, as well as their metabolites over time, were investigated by metabolomics technology. Most metabolites of nucleotides were highly accumulated in the intracellular environment after the mid-exponential phase. Increases in the intracellular unsaturated fatty acids and N-acetyl-glucosamine and N-acetyl-muramoate recycling provided potential evidence that cell envelope remodeling occurred after the mid-exponential phase. At the later fermentation stages, potentially functional metabolite produced by glycine was highly accumulated in the intracellular environment. Additionally, potential toxic metabolites produced by phenylalanine and tyrosine could not be excreted into the extracellular environment in a timely basis. The accumulation of large amounts of these metabolites might be the primary cause of the overconsumption of amino acids and influence the growth of *S. thermophilus*.

## Introduction

*Streptococcus thermophilus* is widely used as a starter in the manufacturing of fermented dairy products and is the second most important species of industrial lactic acid bacteria (LAB) after *Lactococcus lactis* (Ramya et al., 2010). Currently, Direct Vat Set cultures are the most common mode of production in the yogurt fermentation industry (Ma et al., 2013). High-cell-density culturing is one of the key steps that pivotally influences the quality of the starter (Buckenhüskes, 1993). Lactic acid starters are currently produced using pH-controlled pure cultures, of which pH is maintained at an optimal value by continuously adding sodium hydroxide into the culture. Compared to acidic fermentations, pH-controlled cultures led to higher growth rate and final biomass as a result of the lower level of non-dissociated lactic acid in the culture medium (Rault et al., 2009). *S. thermophilus* generates lactic acid as one of the metabolic end-products. The accumulation of sodium lactate results in increases in the extracellular osmotic pressure, which ultimately causes growth arrest in *S. thermophilus* (Huang et al., 2016; Ai et al., 2017). Apart from lactic acid, LAB can generate a number of metabolites with the potential to influence their growth. Particular groups of secondary metabolites can regulate the gene expression of cell-cell communication (Keller and Surette, 2006). Some antimicrobial compounds can suppress the growth of LAB (Van de et al., 2001). Furthermore, the biosynthesis of secondary metabolites consumes a great deal of metabolic energy at significant cost to the cell (Briand et al., 2015). Therefore, increasing the knowledge about metabolic profiling and the changes of key metabolites during the high-density culture of *S. thermophilus* can potentially be beneficial for yield improvements in industrial processes.

Several studies have been conducted to discriminate the metabolome of microbes during different growth phases. The metabolomic profiling of *Escherichia coli* from the exponential phase to the stationary phase was investigated, revealing that the class of phospholipids was the most growth-related metabolite (Takahashi et al., 2008). The metabolic profiling of *Halomonas* sp. during different growth phases characterized the metabolites regulation pattern, which is beneficial to optimize the production of poly(3-hydroxybutyrate) (Jin et al., 2013). Similar study of *Saccharomyces cerevisiae* during different growth stages revealed that the oscillation of metabolites abundance contributed to controlling the temporal regulation of cellular processes (Tu et al., 2007). Additionally, the metabolic profiles in some LAB, such as *Lactobacillus delbrueckii* and *Lactobacillus plantarum*, have also been analyzed. These studies provided the theoretical basis for improving the production of d-lactate and evaluating the fermentative characteristics of starter culture, respectively (Park et al., 2016; Liang et al., 2018). However, few studies have investigated the changes in the metabolome incorporating both the intracellular and extracellular levels in *S. thermophilus*.

The objective of this study is to analyze the intracellular and extracellular metabolite profiles of *S. thermophilus* at different growth stages during pH-controlled batch fermentation using metabolomics technology. Through understanding the metabolic profiles and changes of metabolites in the central metabolic pathways, the growth characteristics of *S. thermophilus* in different stages could be elucidated, which is beneficial for growth maximization during the high-cell-density culture of *S. thermophilus*.

## Materials and Methods

### Strains, Culture Conditions and Fermentation Experiments

**FIGURE 1:**
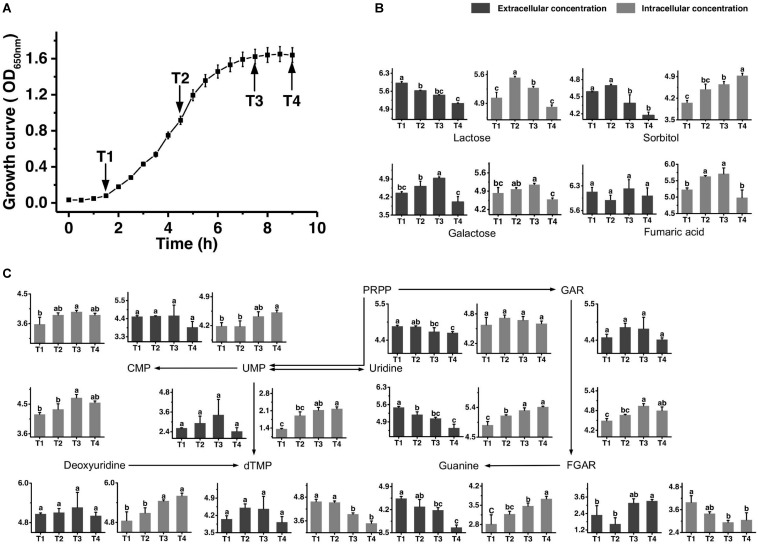
Growth curve of ST-MZ-2 during culture **(A)**. Changes in the concentrations of metabolites associated with the carbohydrate **(B)** and nucleotide **(C)** metabolic pathways in ST-MZ-2 during culture. The *x*-axis displays the sampling time, and the *y*-axis displays the log10 peak area for metabolites. Values are the mean ± SEM, *n* = 3. The bars with different superscripts are significantly different (*p* < 0.05).

*Streptococcus thermophilus* MN-ZLW-002 (ST-MZ-2) was obtained as described in a previous study (Kang et al., 2012). Cells were cultured at 42.5°C for 12 h in chemically defined medium (CDM). The CDM were prepared as described by Letort and Juillard (2010) with some modifications (Supplementary Table S1). The culture was centrifuged (10000 × *g*, 10 min, 4°C); the cells were washed twice with PBS buffer (50 mmol/L, pH 6.5) and then inoculated into a bioreactor (Shanghai Baoxing, Shanghai, China) containing 7 L of CDM. The temperature and rotation speed were fixed at 42.5°C and 200 rev/min, respectively. The pH was maintained at 6.25 through the automatic addition of 1 mol/L NaOH. The pH value was measured by a Delta 320 pH meter (Mettler-Toledo Instruments, United States). Samples were collected from the fermentation at the late-lag (T1), mid-exponential (T2), late-exponential (T3), and stationary (T4) phases (Figure 1A). At the indicated time points, the cultures were centrifuged (12000 × *g*, 4°C, 15 min). The supernatant was snap-frozen in liquid nitrogen and stored at −80°C. The pellet was washed twice with buffer, sub-packed into Eppendorf tubes, and kept at −80°C. Each culture condition was repeated three times.

### Sample Extraction and Preparation

A total of 25 mg samples were dissolved in 200 μl of methanol/water (1/1, v/v) using a TissueLyser (60 Hz) for 5 min (Qiagen, Hilden, Germany). After centrifugation (25000 × *g*, 4°C, 20 min), 300 μL of the supernatant was used for further analysis and a quality control (QC) sample was prepared by mixing equal volumes (200 μL) from each sample. This pooled QC was used to provide a representative “mean” sample containing all the analytes that will be encountered during the analysis. The samples were added to each well of a 96-well plate at 80 μL/well for a total of three plates.

### LC-MS Analysis

All chromatographic separations were performed using an ultra-performance liquid chromatography (UPLC) system (Waters, United States). An ACQUITY UPLC BEH C18 column (100 mm × 2.1 mm, 1.7 μm; Waters) was used for the reversed-phase separation. The column oven was maintained at 50°C. The flow rate was 0.4 mL/min, and the mobile phase consisted of solvent A (water +0.1% formic acid) and solvent B (acetonitrile +0.1% formic acid). Gradient elution conditions were set as follows: 0–2 min, 100% phase A; 2–11 min, 0–100% B; 11–13 min, 100% B; and 13–15 min, 100% A. The injection volume for each sample was 10 μL.

A high-resolution tandem mass spectrometer, SYNAPT G2 XS QTOF (Waters, United States), was used to detect metabolites eluted from the column. The Q-TOF was operated in both positive and negative ion modes. For positive ion mode, the capillary and sampling cone voltages were set at 0.25 kV and 40 V, respectively. For negative ion mode, the capillary and sampling cone voltages were set at 2 kV and 40 V, respectively. The mass spectrometry data were acquired in the Centroid MSE mode. The TOF mass range was from 50 to 1200 Da, and the scan time was 0.2 s. For the MS/MS detection, all precursors were fragmented using 20–40 eV, and the scan time was 0.2 s. During the acquisition, the LE signal was acquired every 3 s to calibrate the mass accuracy. The pooled QC sample was injected 10 times at the beginning of the run to ensure system equilibration and then every 10 samples to further monitor the stability of the LC-MS and assess the quality of the collected samples.

### Data Processing and Statistical Analysis

Raw data from the UPLC/MS were uploaded into Progenesis QI (vision 2.2). Data normalization (with normalization to all compounds) and peak picking (with retention time (RT) and mass to charge ratio (m/z) data pairs) were performed with Progenesis QI which automatically selected the most appropriate “reference” for peak alignment from the QC samples. Data corrections were processed by a quality control-based robust LOESS signal correction (QC-RLSC) algorithm. Ions with a relative standard deviation (RSD) ≤30% in the QC sample analyses were used for further analyses. Principal component analysis (PCA) was performed using SIMCA-P 11.5 software (Umetrics, Umea, Sweden) after the Pareto scaling to investigate the overall metabolome variation caused by the fermentation of ST-MZ-2. PCA analysis was also applied to project the complex data and extract information from the whole metabolic profile in QC sample. In PCA analysis, the tighter of QC samples gathered, the more stable the instrument is, and the better the quality of the collected data. The significance of difference of the metabolite among groups was performed by using Statistix^®^ version 8 (Analytical Software, Tallahassee, FL, United States). A *p*-value < 0.05 was regarded as statistically significant. The identification of metabolites and metabolic pathway analysis were carried out using KEGG^1^.

### Transmission Electron Microscopy (TEM)

Samples were collected from the fermentation at the late-lag (T1), mid-exponential (T2), late-exponential (T3), and stationary (T4) phases. At the indicated time points, the cells were harvested through centrifugation at 8000 r/min for 15 min at 4°C. Cells were diluted in buffer as required after washing twice with PBS buffer (50 mmol l^–1^, pH 6.5). The electron microscopic investigations were carried out using a HITACHI H-7650 electron microscope (Hitachi High-Technologies, Tokyo, Japan). The formvar-coated copper grids were immersed in the bacterial suspension for 10 min; then stained with 2% phosphotungstic acid (PTA) for 5 s after the removal of excess liquid and air drying; the grid was air- dried again before examination.

## Results

**FIGURE 2:**
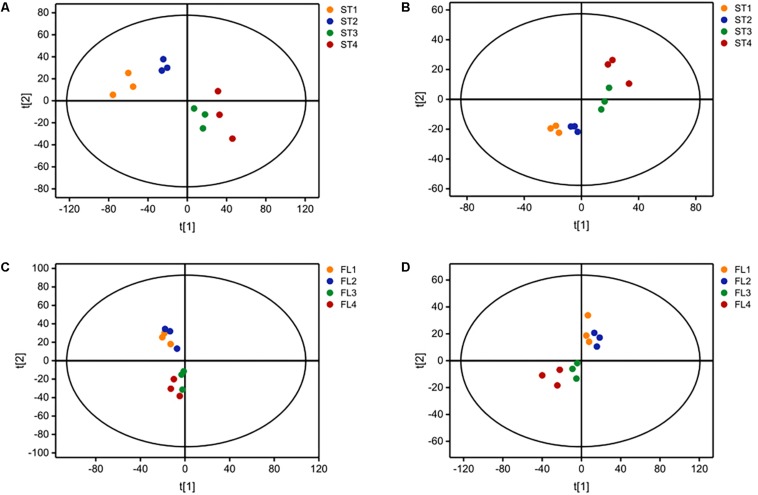
Principal component analysis (PCA) score plot based on LC-MS analysis in **(A)** positive ion mode and **(B)** negative ion mode for ST-MZ-2 intracellular samples (ST); **(C)** positive ion mode and **(D)** negative ion mode for ST-MZ-2 extracellular samples (FL).

The growth curve is shown in Figure 1A. Samples were taken at four different time points representing late-lag (T1), mid-exponential (T2), late-exponential (T3), and stationary (T4) phases. After the peak alignment and the exclusion of ion features with RSD >30% in QC sample analyses, 5594 ion features were imported to the software for PCA analysis. QC analyses were clustered crowdedly in PCA score plot indicating a reliable metabolomics analysis (Supplementary Figure S1). Figure 2 shows the PCA score plot of the metabolic profiles of *S. thermophilus* based on LC-MS analysis in positive and negative ionization modes. The PCA model quality parameters were as follows: *R*^2^*X* > 0.715 and *Q*^2^*Y* > 0.59 for cell metabolites; *R*^2^*X* > 0.51 and *Q*^2^*Y* > 0.332 for medium metabolites. Clear stepwise alterations of intracellular metabolome were observed during the fermentation process from T1 to T4 in both ionization modes. Furthermore, PCA analysis in positive ion mode revealed remarkable difference metabolomic profile from T2 to T3 in intracellular and extracellular environment. Similar differences were observed in PCA analysis in negative ion mode. The information of identified metabolites related to following metabolic pathways were included in Supplementary Table S2.

### Carbohydrate Metabolism

Figure 1B shows the changes in the concentrations of the carbohydrates mentioned below. The extracellular concentration of lactose decreased over time. The intracellular concentration of lactose increased from T1 to T2 and then decreased significantly. The intracellular concentrations of galactose accumulated in the intracellular environment during cell growth, but the accumulated level of galactose decreased after T3. The extracellular concentration of galactose also decreased significantly simultaneously. Sorbitol was highly accumulated in the intracellular environment at T4. The changes in extracellular concentration of sorbitol indicated that this sugar was imported into the intracellular environment, mainly from T2 to T4. Additionally, the intracellular concentration of fumaric acid decreased significantly from T3 to T4.

### Nucleotide Metabolism

Figure 1C shows the metabolic profiles of *S. thermophilus* in the proposed simplified pathway of nucleotide metabolism. The extracellular concentration of PRPP, an extremely important precursor of nucleotide synthesis, decreased during the whole progression of growth. In addition, its intracellular concentration did not change significantly. Regarding purine nucleotide metabolism, guanine provided in the medium was continuously consumed. Guanine and GAR accumulated in the intracellular environment mainly at T3 and T4. The intracellular concentration of FGAR decreased from T1 to T3, whereas extracellular FGAR increased from T2 to T4. The changes in intracellular concentrations of metabolites involved in pyrimidine metabolism (uridine, deoxyuridine, UMP, and CMP) indicated that their synthesis rates were higher than their consumption rates from T2 to T4. Extracellular uridine was greatly consumed throughout the fermentation. However, intracellular dTMP was significantly decreased from T1 to T3.

### Amino Sugar and Nucleotide Sugar Metabolism

**FIGURE 3:**
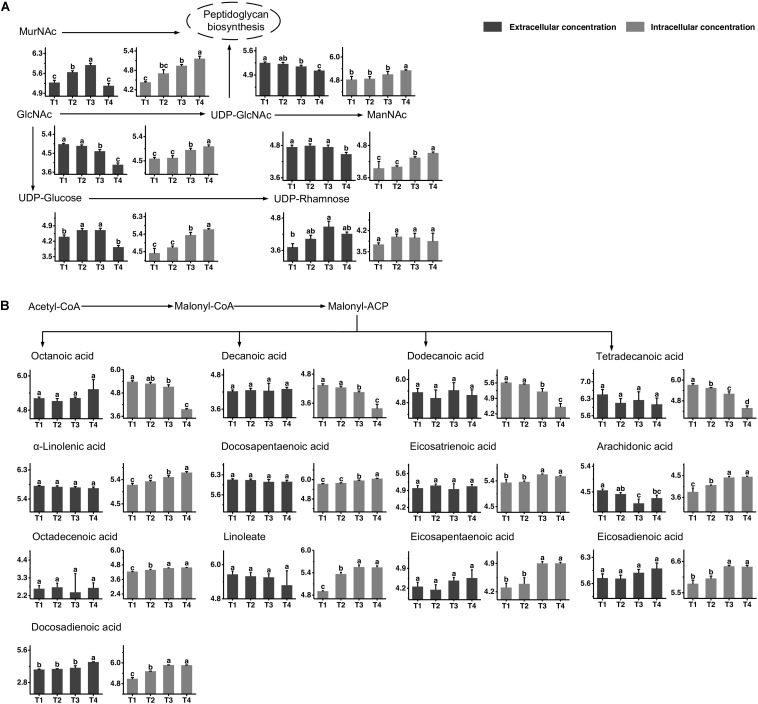
Changes in the concentrations of metabolites associated with the amino sugar and nucleotide sugar **(A)** and fatty acid **(B)** metabolic pathways in ST-MZ-2 during culture. The *x*-axis displays the sampling time, and the *y*-axis displays the log10 peak area for metabolites. Values are the mean ± SEM, *n* = 3. The bars with different superscripts are significantly different (*p* < 0.05).

The metabolic profiles of amino sugar and nucleotide sugar are shown in Figure 3A. The changes in the concentrations of N-acetyl-glucosamine (GlcNAc) and N-acetyl-muramoate (MurNAc) indicated that it might be recycled mainly from T3 to T4 and greatly accumulated in the intracellular environment at T4. The extracellular concentration of N-acetyl-mannosamine (ManNAc) significantly decreased from T3 to T4, and its intracellular concentration increased simultaneously. The changes in intracellular concentrations of UDP-glucose and UDP-GlcNAc indicated that their synthesis rates were higher than their consumption rates from T3 to T4. The extracellular concentrations of UDP-glucose and UDP-GlcNAc significantly decreased from T3 to T4. The change in the extracellular concentration of UDP-rhamnose indicated that it was excreted out of cells mainly from T1 to T3 to maintain a stable intracellular concentration.

### Fatty Acid Biosynthesis

The metabolic profiles of fatty acid biosynthesis are shown in Figure 3B. The changes in intracellular concentrations of octanoic acid, decanoic acid, dodecanoic acid, and tetradecanoic acid indicated a decrease in saturated fatty acid synthesis from T3 to T4. The extracellular concentrations of these fatty acids did not change significantly during growth. For unsaturated fatty acid biosynthesis, the intracellular concentrations of α-linolenic acid and docosapentaenoic acid increased significantly from T3 to T4, indicating that their synthesis rates were higher than their consumption rates. The intracellular concentrations of eicosatrienoic acid, arachidonic acid, octadecenoic acid, linoleate, eicosapentaenoic acid, eicosadienoic acid, and docosadienoic acid were high at T3 and T4. The extracellular concentrations of most unsaturated fatty acids did not change significantly. However, the extracellular concentration of docosadienoic acid increased significantly from T3 to T4, indicating that its excretion rate was higher than its uptake rate in this period. The extracellular concentration of arachidonic acid decreased significantly from T1 to T3.

### TEM Observations

**FIGURE 4:**
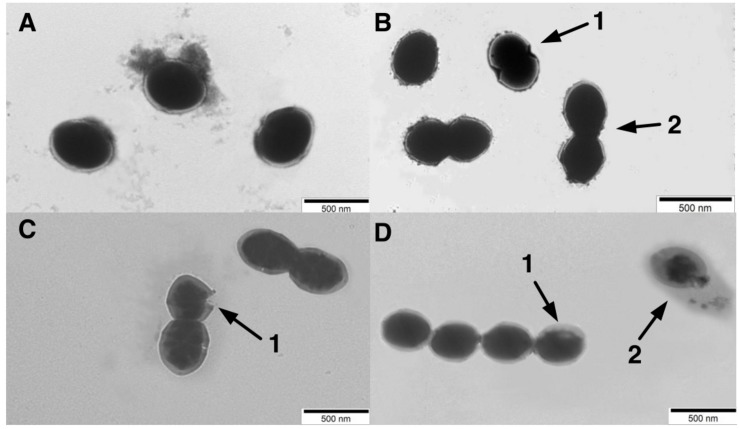
Transmission electronic microscopy (TEM) photograph of ST-MZ-2 at the **(A)** late-lag phase (×5000). **(B)** mid-exponential phase (×5000). 1, the invagination cells. 2, the cell division **(C)** late-exponential phase (×5000). 1, lysis in cell wall and cell membrane. **(D)** stationary phase (×5000). 1, the cytoplasmic membrane was separated from the cell wall. 2, complete lysis of the cell, leakage of cytoplasmic content.

The morphological changes of the *S*. *thermophilus* cells were observed through TEM to investigate the cell metabolism. As observed in Figure 4A, most cells were integrated during the lag phase. The invagination (Figure 4B (1)) and division of cells (Figure 4B (2)) mainly occurred during the exponential phase. Separation of cell wall and cell membrane lysis (Figure 4C (1)) cytoplasmic membrane from the cell wall (Figure 4D (1)) and cytoplasmic content leakage (Figure 4D (2)) were observed during later fermentation stages.

### Amino Acid Metabolism

**FIGURE 5:**
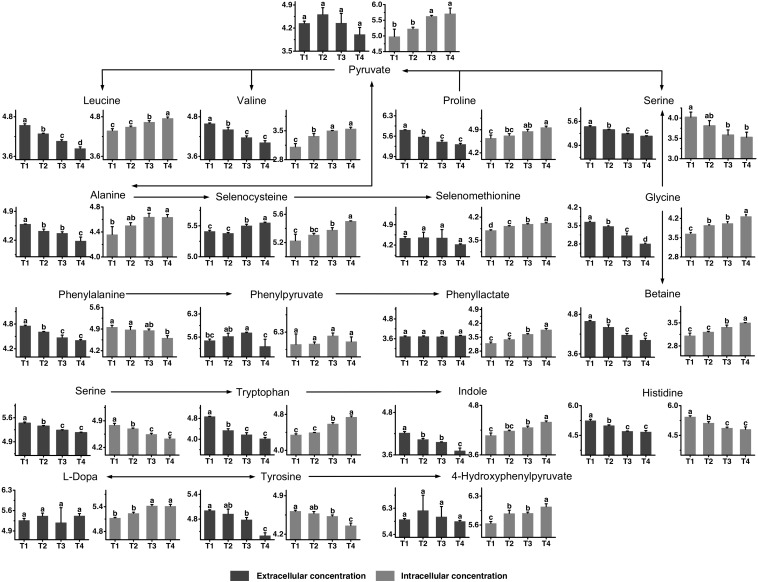
Changes in the concentrations of metabolites associated with the proline, glycine, leucine, valine, histidine, phenylalanine, tyrosine, and tryptophan metabolic pathways in ST-MZ-2 during culture. The *x*-axis displays the sampling time, and the *y*-axis displays the log10 peak area for metabolites. Values are the mean ± SEM, *n* = 3. The bars with different superscripts are significantly different (*p* < 0.05).

The metabolic profiles of proline, glycine, leucine, valine, histidine, phenylalanine, tyrosine, and tryptophan, as well as their metabolites are shown in Figure 5. All amino acids provided by the medium were generally consumed to different extents, although not to the same extend. The consumption of proline, valine, histidine, phenylalanine, and tryptophan seemed to flatten out at the later fermentation stages, while other amino acids, such as glycine, leucine, and tyrosine were continuously consumed throughout the fermentation time. Betaine, the functional metabolite of glycine, was highly accumulated in the intracellular environment from T2 to T4 and its extracellular concentration decreased simultaneously. The changes in intracellular concentrations of valine and leucine indicated that their synthesis rates were higher than their consumption rates from T2 to T4. The intracellular concentrations of selenocysteine and selenomethionine increased significantly from T3 to T4. Meanwhile, extracellular selenocysteine increased significantly, showing that selenocysteine could be excreted to the extracellular environment. The intracellular and extracellular levels of histidine decreased from T1 to T3, indicating that *S. thermophilus* had a greater demand for histidine. The intracellular concentration of phenylalanine decreased from T1 to T4. The extracellular concentration of phenylpyruvate decreased significantly after T3. Phenyllactate was potential toxic metabolites of phenylalanine. The intracellular concentrations of phenyllactate were high at T4. The intracellular concentration of tyrosine decreased significantly after T3. For the metabolites of tyrosine, 4-hydroxyphenylpyruvate and L-Dopa, the intracellular concentrations were high at T3 and T4. The extracellular concentrations of metabolites of tryptophan, such as indole, decreased over time. In addition, the intracellular concentrations of tryptophan and its metabolites were high at T3 and T4. These metabolites might be highly absorbed into the intracellular environment from the extracellular environment from T2 to T4.

**FIGURE 6:**
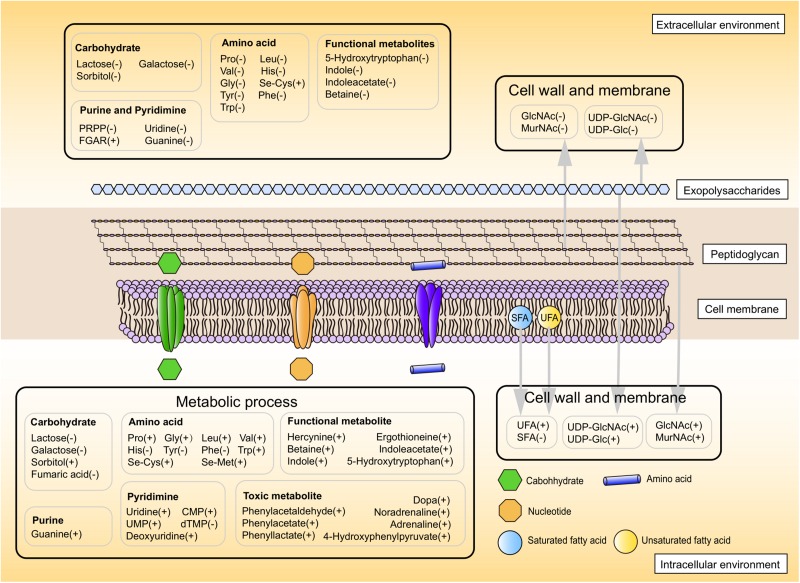
The proposed metabolic profile model of ST-MZ-2 after the mid-exponential phase. Increased and decreased levels are indicated by plus symbol “+” and minus symbol “−,” respectively.

Overall, these results suggested a model for physiological changes during the shift from the mid-exponential growth phase to the stationary growth phase (summarized in Figure 6). Choosing alternative carbon sources, remodeling cell envelope and regulating amino acids and nucleotides metabolic processes were induced upon entry into the later fermentation stages.

## Discussion

The metabolism of other carbohydrates is usually depressed in bacteria if carbohydrates that are readily utilized are present (Görke and Stülke, 2008). Low energy production caused by the exhaustion of extracellular lactose led *S. thermophilus* choose alternative energy sources such as sorbitol. Gene encoding α-galactosidase which catalyzes the formation of sorbitol from galactose was detected in *S. thermophilus* (Donkor et al., 2007). Sorbitol can protect cells against oxidative damage by scavenging off free reactive oxygen radicals (Santivarangkna et al., 2006). In addition to contributing to producing energy, the facilitated influx of sorbitol could be involved in extending the survival of *S. thermophilus* in the stationary phase. Since the TCA cycle is interrupted or not present, *S. thermophilus* needs to utilize alternative ways to synthesize fumaric acid. In *S. thermophilus*, the transcript level of gene *argH* encoding argininosuccinate lyase, which catalyzes the formation of fumaric acid from aspartate, decreased significantly after the late-exponential phase (Qiao et al., 2018). This indicates that the low intracellular level of fumaric acid at the stationary phase is caused by down-regulation of the gene *argH*.

The changes in the intracellular concentrations of most metabolites of purine after the mid-exponential phase indicated that the cells were accumulating purine for DNA and RNA synthesis during this period. Previous studies reported that genes and enzymes involved in the *de novo* purine biosynthetic pathway increased during the exponential phase in *S. thermophilus* (Derzelle et al., 2005), as suggested by the above pathway analysis with metabolomics in the present study. PRPP in the medium was found which in most cases is not present in extracellular medium. The present result did not exclude the possibility of leakage of intracellular metabolites. The autolysis of LAB mainly occurred during the shift from the late-lag phase to the mid-exponential phase and the shift from late-exponential phase to stationary phase (Fernández Murga et al., 1995; Kang et al., 1998). The autolysis of cell led to the leakage of intracellular metabolites, which is a normal physiological phenomenon.

*Streptococcus thermophilus* could modulate the synthesis of peptidoglycan to adapt to challenging environmental conditions in the stationary phase. GlcNAc and MurNAc, the components of the glycan chain of peptidoglycan, were recycled in the stationary phase. The peptidoglycan recycling provides a benefit for the survival fitness of *S. thermophilus* in the stationary phase through being preferentially utilized for cell wall synthesis (Borisova et al., 2016). The recycling and accumulation of ManNAc in the stationary phase could promote the production of sialic acid in *S. thermophilus*. In bacteria, sialic acid can serve as a source of carbon and nitrogen and as a source of amino sugars to synthesize the cell wall (Vimr et al., 2004). UDP-GlcNAc and UDP-glucose are precursors for the synthesis of EPS in *S. thermophilus* (Wu et al., 2014). EPS related-genes in the cluster, *epsA* to *epsM*, were higher transcripted at the stationary phase compared to the earlier growth phases in *S. thermophilus* (Sieuwerts et al., 2010), explaining the metabolic changes of UDP-GlcNAc and UDP-glucose in the present study. EPS production is thought to be an adaptive response to osmotic stress or acid stress in LAB (Caggianiello et al., 2016). Therefore, the changes in the concentrations of these UDP-sugars in the stationary phase can potentially promote the synthesis of EPS, enhancing resistance against environmental stresses. The occurrence of autolysis was mainly observed in the stationary phase with TEM. Autolysis was triggered under unfavorable environmental conditions (Soda et al., 1995). Although autolysis is not a common phenomenum in *S. thermophilus* (Arioli et al., 2018), the autolytic behavior occurred under lactose limitation (Thomas and Crow, 1983), consistent with the present results. The translational levels of muramidase, endopeptidase, and N-acetyl-muramoyl-L-alanine amidase, which promotes peptidoglycan autolysis, increased significantly during the late-exponential phase in *S. thermophilus* (Qiao et al., 2019). These previous results explained the occurrence of peptidoglycan autolysis in the present study. The modulation of a series of metabolites in cell metabolism could be related to the autolysis processes, which triggered by the environmental stresses.

The fatty acid composition was modulated to achieve a higher ratio of unsaturated/saturated fatty acids after the mid-exponential phase in this study. The up-regulation of the ratio of unsaturated/saturated fatty acids is a response to environmental stresses in bacteria (Guerzoni et al., 2001; Wu et al., 2012). The proportion of oleic acid increases to resist osmotic stress in *L. lactis* and unfavorable growth conditions in *S. thermophilus* (Guillot et al., 2000; Beal et al., 2001). The modulation and incorporation of oleic acid and linoleic acid favor acid and oxidative stress tolerance in *Lactobacillus helveticus* (Montanari et al., 2010). The accumulation of arachidonic acid in the intracellular environment plays roles in signaling and cell regulation (Brash, 2001). The importances of the modulation of the peptidoglycan layer and the ratio of unsaturated/saturated fatty acids can be specifically linked to stress conditions during the later stages of fermentation.

Proline and glycine are two types of representative osmoprotectants (Yang et al., 2017). Betaine, the metabolite of glycine, also acts as an effective osmoprotectant in *Lactobacillus salivarius* and *Lactobacillus buchneri* (Sheehan et al., 2006; Louesdon et al., 2014). *S. thermophilus* is currently produced in cultures in which the extracellular pH is controlled at 6.25 through the continuous addition of sodium hydroxide. The previous study demonstrated that the extracellular concentration of sodium lactate in ST-MZ-2 increased from 2.61 to 47.67 mmol/L during the pH-controlled fermentation (Liu et al., 2018). The gradual accumulation of extracellular sodium lactate was an important factor for osmotic stress in *S. thermophilus*. The consumption of these two amino acids and the changes in the concentration of betaine after the mid-exponential phase could be related to osmotic pressure regulation. Selenomethionine and selenocysteine can cause toxicity through being randomly assimilated into proteins (Zhou et al., 2000). The increases in intracellular selenomethionine and selenocysteine most likely depress the growth of *S. thermophilus*. The excretion of selenocysteine could be a response to toxicity. The present results indicated that *S. thermophilus* had a greater requirement for histidine. It was reported to be auxotrophic for glutamate, cysteine, methionine, and histidine in *S. thermophilus* (Arioli et al., 2007), supporting the present result. The changes in histidine concentration could be related to signal transduction in response to environmental changes (Mascher et al., 2006).

Most metabolites of phenylalanine possess antimicrobial activity. Gene coding for aspartate aminotransferase involved in the formation of phenylpyruvate from phenylalanine was detected in *S. thermophilus* (Peralta et al., 2016). Phenylpyruvate converts to phenyllactate by the catalyzing of hydroxyphenylpyruvate reductase in bacteria, which could be linked to potentially biosynthesis pathway of phenyllactate in *S. thermophilus* (Serres et al., 2001). Phenyllactate had a relatively broad antibacterial spectrum and a significant bacteriostatic effect on both gram-positive and gram-negative bacteria (Li et al., 2007). Tyrosine can be converted to a biogenic amine via the tyrosine decarboxylase activity of LAB (Christensen et al., 1999). In this study, tyrosine was highly consumed, consistent with the previous work (Liu et al., 2018). The consumption of tyrosine could be associated with regulation of intracellular pH. Aspartate aminotransferase can catalyze the formation of 4-hydroxyphenylpyruvate from tyrosine in *S. thermophilus* (Peralta et al., 2016). Gene *pgeF* encoding polyphenol oxidase which catalyses the formation of L-Dopa from tyrosine was detected in bacteria (Beloqui et al., 2006). This could be the pathway potentially involved in L-Dopa synthesis. The metabolites of tyrosine, 4-hydroxyphenylpyruvate and L-Dopa are toxic for microorganisms due to the non-repairable damage by reacting with DNA, proteins and membranes (Dean et al., 2001). Excessive accumulation of 4-hydroxyphenylpyruvate is capable of inhibiting the growth of the strains due to its toxicity (Ferreira et al., 2006). The accumulation of toxic metabolites of tyrosine and phenylalanine could be harmful to the growth of *S. thermophilus*. Tryptophan and its derivatives exhibit antioxidant activity through the electron transfer of nitrogen atoms (Sviardlou et al., 2017). Tryptophanase catalyzes the formation of indole from tryptophan in bacteria, which could be the potentially biosynthesis pathway of indole (Kogan et al., 2015). Some bacteria use indole as a signal molecule to combat osmotic stress and to regulate biofilm-promoting factors (Martino et al., 2003; He et al., 2017). The changes in the concentrations of indole in both intracellular and extracellular environments could be related to their functional properties.

Most markedly growth phase-dependent changes at the metabolic level occurred from the mid-exponential to the stationary growth phase in this study. A series of bacterial stress responses could be activated by reduced nutrient levels and harsher conditions after the exponential phase (De and Gobbetti, 2004). At the later fermentation stages, the cell envelope was modified by modulating of the components of the peptidoglycan layer and by increasing the ratio of unsaturated fatty acids. The distinct carbohydrate utilization and cell envelope modification indicated that *S. thermophilus* can adapt to changes in environmental conditions through multiple mechanisms. Previous studies have shown that the consumption of amino acids exceeded the amounts necessary for the growth of *L. lactis* and *S. thermophilus*, implying a significant nitrogen wasting (Lahtvee et al., 2011; Hong et al., 2015; Huang et al., 2016). The overconsumption of amino acids could be related to the production of large amounts of useless or harmful metabolites. The harmful metabolites could not be removed by the bacteria, indirectly blocking cell proliferation (Van de et al., 2001). As shown in the present study, most potentially harmful metabolites of phenylalanine and tyrosine cannot be excreted in a timely basis. The excessive accumulation of these metabolites in the intracellular environment might be an important factor for the termination of *S. thermophilus* growth. Potentially functional metabolites of glycine and tryptophan with similar biological functions were highly accumulated in the intracellular environment in the late fermentation stage. The metabolites of tryptophan in the extracellular environment were reabsorbed into the intracellular environment, which can be linked to resist environmental stresses. Therefore, supplementing of these amino acids and reducing nutrient waste by controlling ratios of nutritional components in the growth media could aid the growth of *S. thermophilus*.

## Conclusion

Changes in metabolites in the intracellular and extracellular environments of *S. thermophilus* over time were determined in this study. Carbohydrates, nucleotides, amino sugars, nucleoside sugars, fatty acids, amino acids, and their metabolites were significantly changed during pH-controlled batch fermentation. Currently, the roles of many metabolites mentioned above and the validation of the proposed pathway at the molecular level in *S. thermophilus* are still limited. However, the results presented here together with future studies involved in regulating related metabolites during high-density culture will be instrumental for understanding the growth characteristics of *S. thermophilus*.

## Data Availability Statement

All datasets generated for this study are included in the article/Supplementary Material.

## Author Contributions

YQ and ZF conceived the presented idea. YQ wrote the manuscript with support from ZF, GL, XL, YQ, XF, and YZ processed the experimental data and performed the analysis. MA and LM supervised the experiment. All authors discussed the results and commented on the manuscript.

## Conflict of Interest

The authors declare that the research was conducted in the absence of any commercial or financial relationships that could be construed as a potential conflict of interest.

## Footnotes

1 https://www.genome.jp/kegg/

## Supplementary Material

FIGURE S1 Principal component analysis (PCA) score plot of intracellular (ST) and extracellular samples (FL) and QC samples in **(A)** positive ion mode and **(B)** negative ion mode.

TABLE S1 Composition of the complete chemically defined medium.

TABLE S2 List of identified metabolites in ST-MZ-2 during culture based on LC-MS analysis.
