# Supplementary figures and images for: Metabolic Pathway Profiling in Intracellular and Extracellular Environments of Streptococcus thermophilus During pH-Controlled Batch Fermentations
Source: Front Microbiol. 2020 Jan 21;10:3144. doi: 10.3389/fmicb.2019.03144 (PMC6990133; doi:10.3389/fmicb.2019.03144)

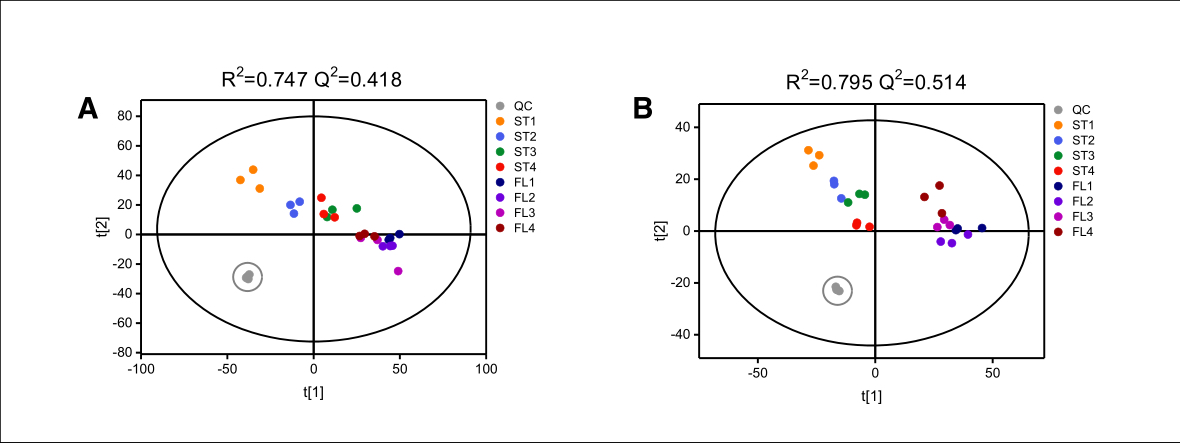

Supplement: FIGURE S1 — Principal component analysis (PCA) score plot of intracellular (ST) and extracellular samples (FL) and QC samples in (A) positive ion mode and (B) negative ion mode. [file Image_1.JPEG]
